# Editorial: Automated vehicles: intelligent decision-making, trajectory planning, and chassis execution

**DOI:** 10.3389/frobt.2024.1464199

**Published:** 2024-09-16

**Authors:** Shuo Cheng, Liang Li, Yong Lei, Xin Xia, Chen Lv

**Affiliations:** ^1^ Institute of Industrial Science, The University of Tokyo, Tokyo, Japan; ^2^ School of Mechanical and Aerospace Engineering, Nanyang Technological University, Singapore, Singapore; ^3^ School of Vehicle and Mobility, Tsinghua University, Beijing, China; ^4^ School of Mechanical Engineering, Zhejiang University, Hangzhou, China; ^5^ Department of Civil and Environmental Engineering, University of California, Los Angeles, CA, United States

**Keywords:** objective detection, visual perception, coverage path planning, collision avoidance, stability control, electric braking control, autonomous vehicles

## 1 Summary

With the rapid development of artificial intelligence and advanced control, automated vehicles (AVs) have attracted extensive interest from both academia and industrial sectors, promising safer, more efficient, and seamlessly integrated mobility solutions. Substantial investigations have been reported for decades, which cover all aspects of autonomous driving, however, we argue that there is still a long path to go for the mass deployment of AVs. To perform autonomous driving tasks in complex and dynamic traffic situations, AVs must perceive accurately the surrounding traffic scenarios in real time, make feasible decision-making about what actions should be taken, plan a safe and collision-free trajectory, and execute the required control commands precisely by chassis actuators. Furthermore, these subtasks must be implemented on the premise of ensuring vehicle handling stability. All procedures still face various open challenges to be solved, for instance, three-dimensional object detection, fast and optimal path planning, coordination of collision avoidance and stability control, and control of electric hydraulic braking system, etc. In the future, research dedicated to autonomous driving technologies will have to concentrate on these crucial aspects, striving to overcome existing technical challenges and pave the way for the large-scale launch of AVs.

This Research Topic aims to provide a platform for researchers to further investigate related issues of autonomous driving and publish their latest research achievements. Organized under the section “Robotics Control Systems” within Frontiers in Robotics and AI, this Research Topic has published four articles. All the accepted papers are summarized as follows.

Autonomous driving functions can only perform effectively with reliable perception and whole-scale environmental awareness. Contreras et al. investigate the state-of-the-art (SOTA) three-dimensional object detection studies utilizing monocular and stereo vision. Prevailing datasets and evaluation metrics are introduced to facilitate the performance evaluation of SOTA detection methods. Generally, three types of detection methods are involved, model-based and geometrically constrained approaches, end-to-end learning methods, and some hybrid methods. After providing a thorough survey of existing detection approaches, the authors discuss current research gaps and bring up prospects for future research.

The article (Champagne Gareau et al.) aims to solve the discrete grid-based coverage path planning problem. An iterative deepening depth-first search method is developed as a baseline approach. The authors further design two branch-and-bound strategies based on loop detection and an admissible heuristic function to enhance the performance of the baseline method. They carry out various experimental tests including simple shapes, random walks, random links, coast-like, labyrinth, and wide-labyrinth grids, and all those results demonstrate the proposed path planning method can achieve optimal performance.

As we all know, vehicle dynamics has strong nonlinear characteristics, especially when a car operates at its handling limits. A vehicle must perform emergency steering maneuvers when encountering some risks. Thus, collision avoidance and handling stability are the two critical issues when an AV operates in an emergency, which typically occurs within a short timeframe and demands significant actuator inputs. The research work (Liu et al.) proposes a collision-avoidance and handling stability coordination method. Both vehicle kinematics and vehicle dynamics are involved in the path-planning strategy in emergencies. The authors design a linear quadratic regulator-based lateral control method for optimizing steering wheel angles. Then, an adaptive model predictive control algorithm and four-wheel braking force distribution strategy are developed to coordinate handling stability and collision avoidance. This work provides a comprehensive framework that combines path planning and control strategies to navigate dangerous scenarios with stability and agility.

The article (Mei et al.) concentrates on the electric braking system, which is one of the most significant bases for autonomous driving systems. The primary challenge in electric hydraulic braking control is to accurately perform position and pressure control. First, the authors introduce a new flow model and divide the whole braking system into three switchable subsystems. Then, based on these subsystems, three corresponding model predictive controllers are designed to construct the switchable control approach. The proposed method improves control accuracy by 22.6% and reduces the response delay by 0.085s.

In this special Research Topic, we explore the intricate domains of environmental perception, trajectory planning, and chassis execution. Each article in this Research Topic represents a significant contribution to the ongoing development of AV technologies, providing insights, innovations, and solutions to facilitate the deployment of AVs. We appreciate all the authors for their valuable contributions to advancing the frontiers of AV technologies. We are on the brink of a new era in transportation to revolutionize human mobility for a safer, and more sustainable future. Finally, we invite readers to delve into the research achievements and insights within these pages, and we hope this Research Topic will motivate new outcomes for the launch of AVs.

